# Empathy with nature promotes pro‐environmental attitudes in preschool children

**DOI:** 10.1002/pchj.735

**Published:** 2024-02-13

**Authors:** Yun Li, Yajun Zhao, Qun Huang, Jiyan Deng, Xueer Deng, Jingguang Li

**Affiliations:** ^1^ College of Teacher Education Dali University Dali China; ^2^ College of Culture, Tourism, and Health Care Chongqing City Vocational College Chongqing China; ^3^ School of Education and Psychology Southwest Minzu University Chengdu China; ^4^ College of Liberal Arts Wenzhou‐Kean University Wenzhou China

**Keywords:** early childhood environmental education, empathy with nature, preschool children, pro‐environmental attitudes

## Abstract

This study aimed to explore whether empathy with nature (i.e., the tendency to understand and share the emotional experience of the natural world) contributes to pro‐environmental attitudes in early childhood. In a correlational investigation (Study 1), 138 (*M*_age_ = 57.78 months) preschool children completed a battery of tasks to measure their pro‐environmental attitudes, empathy with nature, and empathy with humans. We found that empathy with nature positively predicts pro‐environmental attitudes, even beyond the predictive power of empathy with humans. In a quasi‐experimental investigation (Study 2), 46 children from two parallel classes in the same preschool were recruited as the intervention (*n* = 23, *M*_age_ = 66.74 months) and control *(n* = 23, *M*_age_ = 67.61 months) groups. An intervention session that aimed to induce empathy with nature was applied to the intervention group, whereas an active control teaching session was applied to the control group. After the intervention, the intervention group demonstrated greater pro‐environmental attitudes than did the control group. Together, our studies provide converging evidence that empathy with nature promotes pro‐environmental attitudes in early childhood, further implying the value of integrating empathy with nature in early childhood environmental education.

## INTRODUCTION

*Animals suffer as much as we do. True humanity does not allow us to impose such sufferings on them […] Until we extend our circle of compassion to all living things, humanity will not find peace.*

*By Albert Schweitzer (* *1969* *), a humanitarian and Nobel Peace Prize laureate.*

Pro‐environmental attitudes are the cognitive basis of actual pro‐environmental behaviors (Stern, 2000), which further determine the health of our planet. However, pro‐environmental attitudes vary considerably across individuals from childhood (Musser & Diamond, 1999) to adulthood (Milfont & Duckitt, 2010), and some individuals do not recognize the importance of environmental protection. Consequently, identifying the factors that influence various pro‐environmental attitudes is a critical task for environmental researchers (e.g., Gifford, 2014; Gong et al., 2023; Ji et al., 2023; Tian et al., 2022). Given that early childhood is a particularly crucial period for developing environmental attitudes and literacy (North American Association for Environmental Education, 2010), identifying such influential factors in early childhood is especially important and may inspire practitioners to design effective environmental education programs (Ardoin & Bowers, 2020; Güler Yıldız et al., 2021) for children.

In the past two decades, environmental psychologists have demonstrated that individual environmental attitudes and efforts are influenced by one precious quality possessed by human beings: empathy. Empathy is defined as “the drive or ability to attribute mental states to another person/animal, and entails an appropriate affective response in the observer to the other person's mental state” (Baron‐Cohen & Wheelwright, 2004). A number of studies have revealed that a higher score for trait‐level empathy is associated with stronger pro‐environmental attitudes and behaviors (Czap & Czap, 2010; Ienna et al., 2022; Moore & Yang, 2020; Schultz, 2001; Sharma & Christopoulos, 2021; Tam, 2013). Furthermore, some scholars believe that the frequently observed positive association between Big Five agreeableness and pro‐environmental attitudes/behaviors is driven by empathy (Soutter et al., 2020), one facet of agreeableness.

Although the typical objects of the empathic process are human beings, they can also be non‐human living beings. Correspondingly, Tam (2013) explicitly distinguishes two forms of empathy: *empathy with humans* and *empathy with nature*. He defined empathy with nature as “the understanding and sharing of the emotional experience, particularly distress, of the natural world (p. 93)” and developed a questionnaire to measure trait‐level empathy with nature (sample item: “I can very easily put myself in the place of the suffering animals and plants (p. 96)”) in adult samples. Tam found that these two kinds of empathy were only weakly correlated (see also similar findings on the relationship between empathy with humans and empathy with animals in Gómez‐Leal et al., 2021, Paul, 2000, and Taylor & Signal, 2005). More importantly, Tam found that trait‐level empathy with nature positively contributed to conservation behavior, even beyond the variance explained by trait‐level empathy with humans. By testing the adolescent or adult population, Tam's (2013) correlational findings have been directly or conceptually replicated by other researchers (Kim & Cooke, 2021; Liu, 2023; Yin et al., 2021; Zong & Wang, 2017).

Furthermore, scholars have used the experimental approach to study the relationships between empathy with nature and pro‐environmental attitudes and behaviors. That is, experimentally inducing empathy with nature could enhance pro‐environmental attitudes as well as behaviors (Berenguer, 2007; Berenguer, 2010; Jing et al., 2022; Schultz, 2000; Sevillano et al., 2007; Wang, Sheng, et al., 2023). For example, Berenguer (2007) found that individuals who adopted the perspective of a suffering bird or tree felt more compassionate and obligated to help it.

In summary, both experimental and correlational evidence convergingly supported the role of empathy with nature in “green” attitudes and behaviors. This evidence has led some scholars to believe that empathy with nature is vital in building individuals' motivation to conserve the environment and enhance sustainability (see Brown et al., 2019, for a review). However, previous studies have tested only the relationship between empathy with nature and pro‐environmental attitudes in adolescents and adults, predominantly in university students. There is a lack of studies on the period of early childhood, but such exploration is necessary and valuable. Theoretically, investigating whether empathy with nature contributes to early environmental awareness (Musser & Diamond, 1999) may help uncover how children's environmental thoughts originate. Although previous studies have demonstrated that children's pro‐environmental attitudes can be influenced by external factors, such as family, school, media, role models, and exposure to nature (e.g., Eagles & Demare, 1999; Ebersbach & Brandenburger, 2020; Simsar, 2021; Soga et al., 2016), relatively few studies have explored the influence of children's internal thinking on the formation of environmental awareness. Inspired by the studies on empathy with nature in adolescents and adults, we propose that empathy with nature may be an internal factor that shapes children's pro‐environmental attitudes. A qualitative study has provided some preliminary support for this view: environmental awareness exists in children as young as 3 years old, and such awareness may be influenced by empathy for nature (e.g., one child reported that people need to “take care of nature”; Spiteri, 2021); however, it is necessary to verify this hypothesis with quantitative data. Practically, if proving that empathy with nature plays a significant role in pro‐environmental attitudes in early childhood, it may help practitioners design empathy‐based environmental education programs for children in early childhood (see also environmental education courses focusing on empathy with animals for college students in Dolby, 2019).

Therefore, we conducted the present investigation to explore whether empathy with nature contributes to pro‐environmental attitudes in preschool children through two studies. In a correlational investigation (Study 1), we tested the association between empathy with nature and pro‐environmental attitudes in a group of preschool children. In a quasi‐experimental investigation (Study 2), we tested whether inducing empathy with nature would promote pro‐environmental attitudes in preschool children.

## STUDY 1

The primary goal of Study 1 was to examine whether individual differences in empathy with nature are associated with preschool children's pro‐environmental attitudes. If we detected a positive association, we further examined the unique contribution of empathy with nature. This involved controlling for potential confounding factors that also correlate with pro‐environmental attitudes and empathy with nature, such as empathy with humans (Berenguer, 2007; Tam, 2013), and demographic factors, including age, sex, and family socioeconomic status (FSES; Gifford, 2014; Musser & Diamond, 1999; Tam, 2013).

### Materials and methods

#### Participants and procedures

A prior power analysis demonstrated that a sample size of 84 is needed to detect a conventional medium effect size of correlation (*r* = .30; J. Cohen, 1988) with an alpha level = .05 and power = 0.80% using G*Power 3.1 (Faul et al., 2009). However, we recruited more participants, given the exploratory nature of our investigation. The participants included 138 children from the same preschool in Dali, Yunnan Province, China. The ages of the children ranged from 48 to 72 months (*M* = 57.78 months, *SD* = 7.74 months), and 49.28% of the participants were girls. The children completed three tasks that measured their pro‐environmental attitudes, empathy with humans, and empathy with nature. Each child was tested individually in quiet rooms of the children's preschool. In addition, one caregiver of each child (e.g., father or mother) completed an online survey to indicate the child's FSES. The Medical Ethics Committee of Dali University approved the study. We obtained written consent from the children's caregivers and school teachers.

#### Measures

##### Scale of preschool children's pro‐environmental attitudes

**FIGURE 1:**
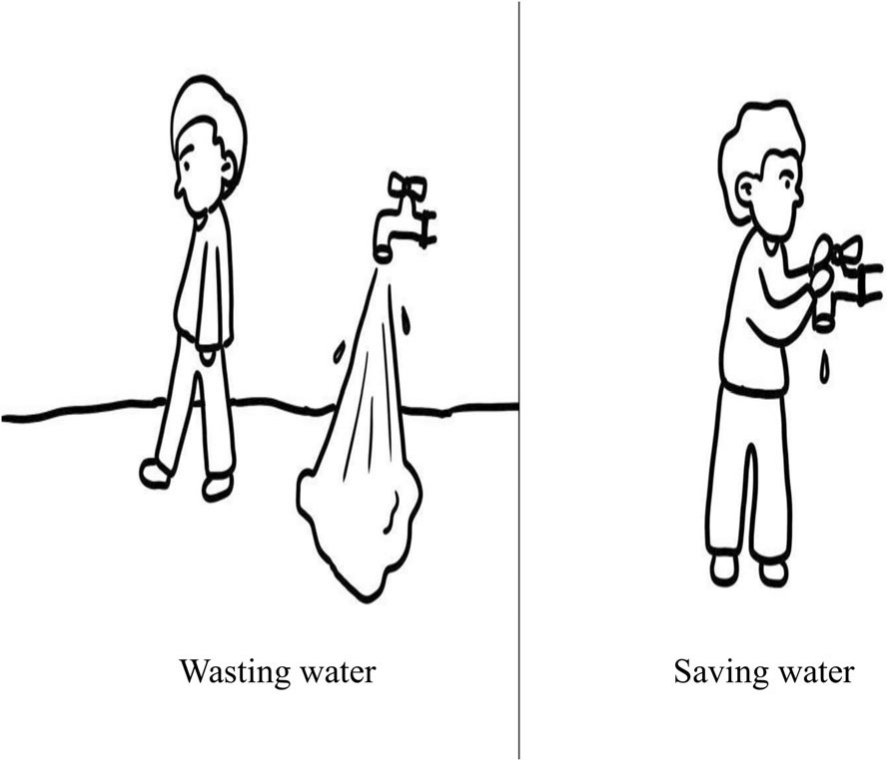
Example stimuli for testing pro‐environmental attitudes. The children were presented with a pair of simple line drawings depicting two alternative behaviors (i.e., pro‐environmental vs. environmentally unfriendly). Meanwhile, the research assistant read the descriptions of the drawings (e.g., “The man washed his hands, but he didn't turn off the water” and “The man washed his hands and turned off the water”). Finally, the research assistant asked the children to indicate their preferences towards the drawings.

Following existing tools in the literature (S. Cohen & Horm‐Wingerd, 1993; Musser & Diamond, 1999; Soydan & Samur, 2014; Wang & Wang, 2016), we developed a scale to measure preschool children's pro‐environmental attitudes based on their preferences for pro‐environmental behaviors in pictorial forms. The scale contains 11 items relating to individuals' attitudes toward the environment, including air pollution, water pollution, noise pollution, soil pollution, animal protection, plant protection, waste disposal, waste classification, water conservation, electricity conservation, and paper saving. For each item, the children were presented with a pair of simple line drawings depicting two alternative behaviors (i.e., pro‐environmental vs. environmentally unfriendly). Moreover, the research assistant read the descriptions of the drawings (e.g., water conservation: “The man washed his hands, but he didn't turn off the water” and “The man washed his hands and turned off the water”; Figure 1). The figures and descriptions of the remaining items are included in the supplementary materials (Figure S1–S10). The research assistant subsequently asked the children to indicate their preferences for the drawings. The research assistant first asked, “Which drawings do you prefer?” The choice of environmentally unfriendly drawing was recorded as 0. If the children chose the pro‐environmental drawing, the research assistant further asked, “Do you like the drawing a little bit or a lot?” The “a little bit” answer was coded as 1, and the “a lot” answer was coded as 2.

Because the scale of preschool children's pro‐environmental attitudes is a newly developed measurement tool, we have examined its psychometric properties. First, to explore the underlying structure of the scale, we performed exploratory factor analysis. The Kaiser–Meyer–Olkin (KMO) measure of sampling adequacy was 0.81, revealing the possibility of common factor(s). Bartlett's tests of sphericity indicated adequate sampling and significant correlations among the observed variables, χ^2^ (55) = 264.97, *p* < .001. All the analyses indicated that the dataset was suitable for factor analysis. Using principal component analysis (PCA), we found that the first three eigenvalues for the dataset were 3.40, 1.18, and 1.07. We performed a parallel analysis to determine the number of factors, which compares the eigenvalues of real data to those of the simulated data (O'Connor, 2000). Specifically, the first three eigenvalues of the simulated data (times of permutation = 1000) were 1.46, 1.34, and 1.23, respectively. The point where the eigenvalues of the real data dropped below the simulated data came after only the first factor. Thus, the dataset demonstrated a single‐factor model (see Table S1 for the promax‐rotated factor loadings of each item for a one‐factor solution in the supplementary materials). Therefore, the total score of all the items was used to index children's pro‐environmental attitudes. Second, we also performed a reliability analysis and found that the scale demonstrated adequate internal reliability, with Cronbach's α = .76 (see Table S2 and S3 for more detailed reliability and item analyses in the supplementary materials).

##### Empathy Continuum Scoring System

We used the Empathy Continuum Scoring System (Strayer, 1993) to evaluate children's empathy with humans and empathy with nature. The Chinese version of the Empathy Continuum Scoring System has been successfully used in Chinese preschool children (Xie, 2017).

The testing procedure involved several steps. First, the research assistant read stories to each child that aimed to evoke empathetic responses. When measuring empathy with humans, the story is about a child's favorite storybook being lost (Script: *Xiao Ming has a special favorite storybook. No matter where he goes, he has to bring his favorite storybook. One day, Xiao Ming took the book to the park to read, halfway to the toilet, and came back to find that the book was missing*). When measuring empathy with nature, the story is about the animals' home in a forest that has been destroyed (Script: *Once upon a time, in a big, beautiful forest, there lived many, many cute little animals. One day, the animals were invited to the city zoo to attend a party. After the animals returned home, they found that their forest was destroyed and bare. Since then, the animals have had no sheltered place*). Second, the research assistant asked the children to briefly paraphrase the story to ensure that they understood the story. Finally, the research assistant asked the children to report their feelings and thoughts about the stories; they were asked: (1) to indicate Xiao Ming's or the animals' feelings (e.g., happy/sad/the same as usual) in the story and to identify the intensity of these feelings as “a little,” “moderate,” or “strongly”; (2) to indicate their own feelings and intensity of feelings; and (3) to explain why they have these feelings.

After the testing procedure, the research assistant coded the children's responses based on the Empathy Continuum Scoring System. The scoring system evaluates both the cognitive and affective components of empathy. For the affective component, children's responses were coded on a 4‐point scale assessing their emotions, ranging from 0 (*child felt no emotion*) to 3 (*child's emotion and intensity is the same as the character's*). For the cognitive components, children's responses were coded on a 4‐point scale assessing their explanation of the feelings, ranging from 0 (*inaccurate identification of character's emotion*) to 7 (*explicit statements of role‐taking*). Finally, the overall empathy score (ranging from 0 to 19) was calculated from the scores of the affective and cognitive components based on the algorithm created by the developer of the scoring system (Strayer, 1993, Table 2, p. 194).

##### Family socioeconomic status

To assess the children's FSES, we asked a caregiver of each child to indicate the rung on a ladder that best represented their overall perception of their FSES (including education, occupational prestige, and income) compared to their local community (Adler et al., 2000). The rungs range from 1 (low FSES) to 10 (high FSES). The Chinese version of the FSES scale has been widely used in Chinese adults (e.g., Wang, Zhao, & Li, 2023).

### Results and discussion

**FIGURE 2:**
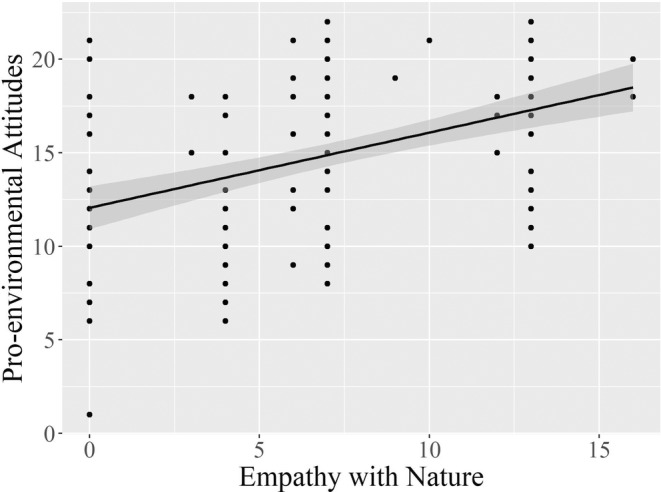
Scatter plot, with a fitted regression line and 95% confidence interval band, depicting a positive linear relationship between empathy with nature and pro‐environmental attitudes in Study 1.

Table 1 presents the means, standard deviations, and correlations for the measures. There were noticeable individual differences in both empathy with nature and pro‐environmental attitudes. Most importantly, empathy with nature was positively correlated with pro‐environmental attitudes (*r* = .46, *p* < .01). That is, children who empathize with nature more strongly have stronger pro‐environmental attitudes (see the corresponding scatter plot in Figure 2).

**TABLE 1:** Means, standard deviations (*SD*), and correlations among the major variables in Study 1.

Variables	Mean	*SD*	1	2	3	4	5
1. Pro‐environmental attitudes	15.06	4.07	–	–	–	–	–
2. Empathy with nature	7.45	4.69	0.46***	–	–	–	–
3. Empathy with humans	6.27	5.18	0.35***	0.49***	–	–	–
4. Age	57.78	7.74	0.39***	0.19*	0.21*	–	–
5. Gender	–	–	0.08	0.11	0.18*	0.09	–
6. Family socioeconomic status	5.52	1.55	0.14	0.03	−0.01	−0.05	−0.04

*Note*: Gender: 1 = boy, 2 = girl.

* *p* < .05;

*** *p* < .001.

As we noted previously, several variables (i.e., empathy with humans and demographic variables) might confound the association between empathy with nature and pro‐environmental attitudes (see also Table 1). Therefore, to test whether the correlation between empathy with nature and pro‐environmental attitudes held when these variables were controlled for, we performed additional hierarchical regression analyses (Table 2). Specifically, the regression model included demographic variables (i.e., age, sex, and FSES) in the first step, empathy with humans in the second step, and empathy with nature in the third step. In the first model, demographic variables explained 18.22% of the variance in pro‐environmental attitudes, and both age and FSES score positively contributed to pro‐environmental attitudes. In the second model, empathy with humans explained additional variance (*ΔR* ^2^ = 7.12%, *p* < .001) in pro‐environmental attitudes beyond the variance explained by demographic variables. In the final model, empathy with nature explained additional variance (*ΔR* ^2^ = 8.84%, *p* < .001) in pro‐environmental attitudes beyond the variance explained by demographic variables and empathy with humans. Therefore, empathy with nature has incremental power for predicting pro‐environmental attitudes beyond other previously established predictors.

**TABLE 2:** Hierarchical regression models for predicting pro‐environmental attitudes in Study 1.

	B (*SE*)	β	*R* ^2^	∆*R* ^2^
Step 1			18.22%***	
Age	0.21 (0.04)	0.39***		
Gender	0.43 (0.64)	0.05		
Family socioeconomic status	0.43 (0.21)	0.17*		
Step 2			25.34%***	7.12%***
Age	0.18 (0.04)	0.34***		
Gender	0.06 (0.62)	0.01		
Family socioeconomic status	0.43 (0.20)	0.16*		
Empathy with humans	0.22 (0.06)	0.28***		
Step 3			34.18%***	8.84%***
Age	0.16 (0.04)	0.31***		
Gender	0.01 (0.58)	0.00		
Family socioeconomic status	0.39 (0.19)	0.15*		
Empathy with humans	0.09 (0.07)	0.11		
Empathy with nature	0.30 (0.07)	0.33***		

*Note*: Gender: 1 = boy, 2 = girl.

* *p* < .05;

*** *p* < .001.

Finally, both empathy with nature (*r* = .19, *p* < .05) and pro‐environmental attitudes (*r* = .39, *p* < .001) were positively correlated with age, suggesting that both constructs were under ongoing development. Therefore, we further asked whether the development of empathy with nature could explain the development of pro‐environmental attitudes. Consequently, we performed a mediation analysis using the “lavaan” package in R (Rosseel, 2012), with reporting of standardized coefficients. After including empathy with nature as an intermediate variable, the associations between age and pro‐environmental attitudes decreased from 0.39 to 0.31, although were still significant (*p* < .001). The 1000 bootstrap simulations further revealed that empathy with nature partly mediated the associations between age and pro‐environmental attitudes: indirect effect = 0.08, 95% CI = [0.01, 0.16], *p* = .04. Together, the development of empathy with nature partly explained the development of pro‐environmental attitudes.

In summary, we confirmed the hypothesis that empathy with nature and pro‐environmental attitudes are positively linked in early childhood, which extends the association from college students to young children (Tam, 2013; Zong & Wang, 2017). The effect size of the association in the present study (*r* = .46) was medium and comparable to that reported in previous studies (Tam, 2013: *r* = .44 for environmental movement support, *r* = .35 for green behavior frequency; Zong & Wang, 2017: *r* = .37 for pro‐environmental behaviors; Kim & Cooke, 2021: *r* = .65 for intention to protect ocean health; Yin et al., 2021: *r* = .53 for environmental citizenship behavior; Liu, 2023: *r* = .36/.45 for environmental attitudes/intentions), suggesting that the degree of contribution of empathy with nature to environmental protection might be similar in both early childhood and adulthood. Furthermore, we confirmed the previous findings that pro‐environmental attitudes increase with age in childhood (Musser & Diamond, 1999; Wang & Wang, 2016) and extend these findings by suggesting that the development of pro‐environmental attitudes in early childhood may be caused by the development of empathy with nature.

## STUDY 2

Next, we explored whether the induction of empathy with nature could enhance preschool children's pro‐environmental attitudes in real education settings.

### Materials and methods

#### Participants

A prior power analysis demonstrated that a sample size of 52 (i.e., 26 for the experimental group and 26 for the control group) is needed to detect a large effect size of group differences (Cohen's *d* = 0.80), which has been reported in similar investigations on adults,^i^ with an alpha level = .05 and power = 0.80%, using G*Power 3.1 (Faul et al., 2009). Based on the power analysis and practical constraints (i.e., the number of students in a class on the day of the experiment), we recruited 46 children from two parallel classes in the senior grade of a preschool in Dali, Yunnan Province, China. Children from one class were randomly assigned to the experimental group (*n* = 23, *M*_age_ = 66.74 months, 12 girls), while children from another class were assigned to the active control group (*n* = 23, *M*_age_ = 67.61 months, 12 girls). There were no significant group differences in age (*t* [44] = 1.33, *p* = .19) or sex. The Medical Ethics Committee of Dali University approved the study, and we obtained written informed consent from all the children's parents and preschool teachers.

#### Procedures

This study contains two stages. In the first stage, by telling and discussing stories presented in picture books, we induced empathy with nature in the experimental group and thinking about friendship in the active control group.^ii^ For each group, the same preschool teacher performed the storytelling for all the children together. In the second stage, we measured children's pro‐environmental attitudes with the same tool used in Study 1 (Cronbach's *α* = .73 for the current dataset). In this phase, the children were tested individually in a quiet area of their preschool by the same research assistant.

Below, we describe the detailed procedures of manipulating empathy with nature in the experimental group and thinking about friendship in the active control group.

##### Inducing empathy with nature

The teacher read a picture book titled *Stranger in the Forest* with the children. In the story, animals live peacefully in their forest until humans arrive. When the human beings cut down all trees, the animals become homeless. After the reading, the teacher asked questions that aimed to induce empathy with the animals. Specifically, the children were asked to identify the animals' emotions and thoughts in the story and report their own emotions and thoughts while listening to the story.

##### Inducing thinking about friendship

The children in the active control group were subjected to a similar procedure of reading a picture book titled *A Pair of Tree Friends in the Forest* and answering related questions. This story is about the “friendship” between two trees. In the beginning, the two trees were neighbors and friends. However, when the two trees were cut down, they lost contact with each other. However, because both trees had a special pattern, they later became blocks and boxes and recognized each other. Therefore, the good friends met happily again. Specifically, the children were asked to identify the trees' emotions, think about the friendship between two trees in the story, and report their own emotions and thoughts while listening to the story.

### Results and discussion

**FIGURE 3:**
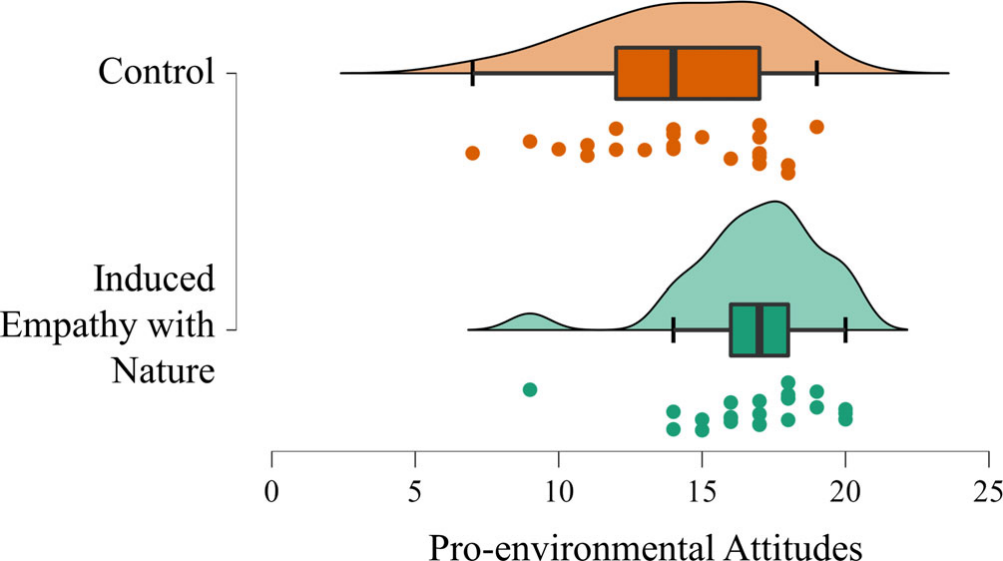
The effect of inducing empathy with nature (versus inducing thoughts about friendship) on pro‐environmental attitudes in Study 2. The results are comprehensively illustrated by a raincloud plot (Allen et al., 2021) that combines a box plot for descriptive statistics, a one‐sided violin plot for probability density, and a cloud of raw data points.

To determine whether the intervention of empathy with nature influences children's pro‐environmental attitudes, we performed an independent *t*‐test to compare the pro‐environmental attitudes between the two groups. As expected, the experimental group (*M* = 16.83, *SD* = 2.46) scored higher on pro‐environmental attitudes than did the active control group (*M* = 14.17, *SD* = 3.19), *t* (44) = 3.16, *p* = .003, Cohen's *d* = 0.94, Hedge's *g* = 0.92 (Figure 3). Therefore, the induction of empathy with nature successfully promotes pro‐environmental attitudes in preschool children, which is consistent with similar intervention effects observed in the adult population (Berenguer, 2007; Berenguer, 2010; Jing et al., 2022; Schultz, 2000; Sevillano et al., 2007; Wang, Sheng, et al., 2023). In addition, the intervention effect in our study is relatively greater than the average intervention effect on pro‐environmental behavior in the literature (averaged Hedge's *g* = 0.45; see Osbaldiston & Schott, 2012, for a meta‐analysis of 87 reports), providing preliminary evidence of the practical significance of the intervention program.

## GENERAL DISCUSSION

In this investigation, we explored the association between empathy with nature and pro‐environmental attitudes among Chinese preschool children. First, we found that individual differences in empathy with nature were positively correlated with pro‐environmental attitudes, even after controlling for empathy with humans and demographic factors. Second, we found that induced empathy with nature is linked to a greater level of pro‐environmental attitudes. Together, these results suggested that empathy with nature may specifically promote preschool children's pro‐environmental attitudes, consistent with similar findings based on adult participants. Furthermore, these findings also highlight the possibility of integrating empathy with nature in early childhood environmental education. Next, we discuss the theoretical implications, practical implications, and limitations of these findings.

### Theoretical implications

Our findings may contribute to the theoretical understanding of pro‐environmental attitudes and behaviors. First, our study revealed that empathy with nature positively predicts individual differences in pro‐environmental attitudes in young children aged 4 to 6 years, and the former's development could account for the latter's development. This evidence complements existing environmental theories, such as the theory of planned behavior (Ajzen, 1991) and value–belief–norm theory (Stern, 2000), which often hypothesize that pro‐environmental attitudes are the starting causes of subsequent pro‐environmental behaviors. Specifically, based on the evidence above, we argue that the origin of pro‐environmental attitudes in early childhood may result from the emergence and development of empathy with nature, although the extant literature has focused mainly on the external factors that shape children's environmental thoughts (Eagles & Demare, 1999; Ebersbach & Brandenburger, 2020; Simsar, 2021; Soga et al., 2016). Certainly, more sophisticated developmental psychology designs and younger participants are needed to examine this hypothesis rigorously.

Second, our study revealed that the influence of empathy with nature on pro‐environmental attitudes in early childhood is beyond the contribution of empathy with humans, which is consistent with similar findings in adults (Tam, 2013). Hence, empathy with nature and empathy with humans exhibit a certain degree of separation during early childhood (see Gómez‐Leal et al., 2021, Paul, 2000, and Taylor & Signal, 2005, for evidence found in adults), which further invites future research into how empathy with nature emerges and develops. Previous findings on the adult population have revealed that stronger connectedness to nature and anthropomorphism are associated with stronger empathy with nature (Fido & Richardson, 2019; Tam, 2013; Zong & Wang, 2017). Therefore, these factors may also contribute to the building of young children's empathetic thoughts towards nature.

Third, our study raises several questions on *how* empathy with nature influences children's pro‐environmental attitudes. Does empathy with nature directly influence pro‐environmental attitudes, or does it influence through certain mediating variables, such as the connection between the self and nature (Schultz, 2000)? How does empathy with nature interact with other external factors (e.g., reward and punishment, role models, and parenting) in shaping children's environmental attitudes? Are the influences of empathy with nature on two types of pro‐environmental attitudes comparable—one towards a specific non‐human entity (e.g., an elephant) and the other towards the habitat of that entity (e.g., a forest)?

### Practical implications

Our findings also have practical implications for early childhood environmental education. First, our study suggested that it is necessary to integrate empathy with nature in early childhood environmental education practices. Two recent systematic reviews on early childhood environmental education did not explicitly mention “empathy with nature” in their papers (Ardoin & Bowers, 2020; Güler Yıldız et al., 2021). Furthermore, the current practice of early childhood environmental education seems action‐oriented: “time in nature” (76%) and “play and movement” (58%) have been found to be the most common themes of early childhood environmental education practices reported in the literature (Ardoin & Bowers, 2020). Compared with action‐oriented practices, educational practices involving empathy with nature may better shape children's intuitive environmental thoughts and feelings based on the evoked empathetic processes. In addition, the effects of intervention based on empathy with nature might be more long‐lasting due to its ability to change children's internal thinking and intrinsic motivation for environmental protection (Darner, 2009).

Second, our study may shed light on contemporary environmental protection topics, such as biodiversity protection and climate change control. As a result of human activities, species are becoming extinct much faster than they were in the past, and biodiversity is rapidly diminishing (Ceballos et al., 2015), which may jeopardize human health and well‐being (Aerts et al., 2018). Although our study did not intentionally focus on endangered animal conservation, the measurement and manipulation of empathy with nature involved an existential crisis of animals. Thus, future studies may test whether inducing empathy with nature is a potentially useful tool for endangered species conservation and biodiversity protection in early childhood. Furthermore, climate change, currently one of the most important environmental crises, has been considered one of the major causes of species extinction (Román‐Palacios & Wiens, 2020). Therefore, if endangered species conservation can be promoted through the induction of empathy with nature, people's efforts to reduce climate change can also be indirectly promoted by this strategy.

Third, our study has demonstrated an easily implementable educational approach targeting environmental protection in preschool settings. Due to its time and resource efficiency, reading picture books that nurture empathy with nature is a more efficient method of environmental education than visiting natural areas and implementing environmental activities. Moreover, in contrast to direct instruction, telling stories is more accessible for young children because they align better with their cognitive development (Altun, 2020). Lastly, narrating the story of animals facing an environmental crisis from an anthropomorphic perspective (i.e., the viewpoint of animals) might be more likely to evoke children's environmental awareness than an objective viewpoint (Williams et al., 2021).

### Limitations

Finally, several limitations of this investigation should be acknowledged and could be addressed in future studies. First, the current investigation only measures pro‐environmental attitudes. However, such attitudes do not guarantee actual pro‐environmental behaviors because of possible environmental attitude–behavioral gaps (Farjam et al., 2019; Redondo & Puelles, 2017; Wyss et al., 2022). Future investigations are needed to explore how empathy with nature influences explicit pro‐environmental behaviors. Second, we only used animals as the objects of empathy with nature, given that empathy with animals plays an influential role in pro‐environmental efforts (Young et al., 2018). However, future investigations may extend the current findings by using other objects of empathy, including other living (e.g., plants) and nonliving (e.g., soils) objects. Finally, the research design of the current study precludes us from making strict causal inferences between the study variables. We collected cross‐sectional but not longitudinal data in Study 1. In addition, Study 2 could only be considered quasi‐experimental because: (1) to better align the study with real teaching settings, we only performed a random allocation of school classes but not individual participants; and (2) to prevent the “pollution” of pre‐test on post‐test of pro‐environmental attitudes across a short time interval, we did not measure these attitudes before the intervention. Future studies may consider using longitudinal and stricter experimental designs.

## CONCLUSION

The current study provides evidence of the relationship between empathy with nature and pro‐environmental attitudes in preschool children. That is, stronger empathy with nature is associated with stronger pro‐environmental attitudes. Moreover, inducing empathy with nature can promote children's pro‐environmental attitudes. Together, our study demonstrated the value of integrating empathy with nature in early childhood environmental education.

## FUNDING INFORMATION

This work was supported by the National Natural Science Foundation of China Grant (32160201) and the Mental Health Education Mentor Team Incubation Project of Yunnan Province awarded to J.L.

## CONFLICT OF INTEREST STATEMENT

The authors have no conflicts of interest to declare.

## ETHICS STATEMENT

The Medical Ethics Committee of Dali University approved the study. We obtained written consent from the children's caregivers and school teachers.

## Supporting information


**Data S1.** Supporting Information.

## Data Availability

Data will be made available on request.

## Endnotes

^i^
Calculated from *t/df* or descriptive statistics (*M/SD*), we found that previous studies on adults generally reported large effect sizes of induction of empathy with nature on various measures of pro‐environmental attitudes: (1) on the willingness to pay a premium for green products, Cohen's *d* = 2.51 (Jing et al., 2022); (2) on moral obligation to help a bird or tree, Cohen's *d* = 0.67 (Berenguer, 2007); and (3) on the number of anthropocentric arguments, Cohen's *d* = 4.20 (Berenguer, 2010). Therefore, in the power analysis, we used the conventional criterion for large effect sizes (Cohen's *d* = .80) proposed by J. Cohen (1988).

^ii^
Suggested by one reviewer, we conducted an additional experiment to check whether empathy manipulation indeed induces empathy with nature. We randomly assigned 140 senior grade children in a preschool from Chongqing city of China to the experimental group (*n* = 70, *M*_*age*_ = 66.86 months, 37 girls) and the control group (*n* = 70, *M*_*age*_ = 67.03 months, 37 girls). Then, we replicated the exact procedure of inducing empathy with nature in the experimental group and thinking about friendship in the active control group as in the formal experiment of Study 2. Finally, we measured children's empathy with nature using the Empathy Continuum Scoring System based on the children's response towards a group of penguins whose habitat was lost (Script: *In the Antarctic, a joyful group of penguins faced a growing threat as small rivers emerged in the ice, intensifying the melting. Contemplating relocation, the penguin family navigated through black water and debris, discovering the impact of the human world with skyscrapers. However, they secured a new home with assistance from other animals. Unfortunately, the joy was short‐lived as the ice in their new abode cracked, leaving them once again without a home*). As expected, an independent *t*‐test demonstrated that the experimental group (*M* = 7.09, *SD* = 4.43) scored higher on empathy with nature than the active control group (*M* = 3.96, *SD* = 4.08), *t*(138) = 4.35, *p* < .001, Cohen's *d* = 0.73. Therefore, the manipulation of empathy with nature in the formal experiment is successful.
