# Supplementary material for: Empathy with nature promotes pro‐environmental attitudes in preschool children
Source: Psych J. 2024 Feb 13;13(4):598–607. doi: 10.1002/pchj.735 (PMC11317190; doi:10.1002/pchj.735)
Supplement: Supplementary file 1 — Data S1. Supporting Information. [file PCHJ-13-598-s001.docx]

**Supplementary Materials**

Table S1. Principal component analysis of the scale of preschool children’s pro-environmental attitudes: Promax-rotated factor loadings (pattern matrix) for a one-factor solution.

| Items | Standardized loadings | Communality variance | Uniqueness variance |
| --- | --- | --- | --- |
| Item 1 (air pollution) | 0.36 | 0.13 | 0.87 |
| Item 2 (water pollution) | 0.46 | 0.21 | 0.79 |
| Item 3 (noise pollution) | 0.57 | 0.33 | 0.67 |
| Item 4 (soil pollution) | 0.38 | 0.14 | 0.86 |
| Item 5 (animal protection) | 0.48 | 0.23 | 0.77 |
| Item 6 (plant protection) | 0.67 | 0.45 | 0.55 |
| Item 7 (waste disposal) | 0.49 | 0.24 | 0.76 |
| Item 8 (waste classification) | 0.51 | 0.26 | 0.74 |
| Item 9 (water conservation) | 0.57 | 0.32 | 0.68 |
| Item 10 (electricity conservation) | 0.58 | 0.34 | 0.67 |
| Item 11 (paper saving) | 0.22 | 0.05 | 0.95 |

Table S2. Detailed reliability statistics of the scale of preschool children’s pro-environmental attitudes.

| raw.alpha | std.alpha | lower.alpha | upper.alpha | G6 | average.r |
| --- | --- | --- | --- | --- | --- |
| 0.76 | 0.77 | 0.70 | 0.76 | 0.77 | 0.23 |

Note: raw.alpha = Cronbach’s α based on row scores; std.alpha = Cronbach’s α based on standardized items; lower.alpha = lower 95% confidence boundary; upper.alpha = upper 95% confidence interval; G6 = Guttman’s Lambda 6 reliability score; average. r = the average inter-item correlation.

Table S3. Item-wise statistics of the scale of preschool children's pro-environmental attitudes.

| Items | Mean | SD | Skewness | Kurtosis | r.raw | r.drop | alpha.drop | average.r.drop |
| --- | --- | --- | --- | --- | --- | --- | --- | --- |
| Item 1 (air pollution) | 1.06 | 0.72 | -0.09 | -1.10 | 0.48 | 0.33 | 0.75 | 0.24 |
| Item 2 (water pollution) | 1.29 | 0.61 | -0.23 | -0.65 | 0.51 | 0.39 | 0.74 | 0.23 |
| Item 3 (noise pollution) | 1.34 | 0.71 | -0.59 | -0.87 | 0.60 | 0.47 | 0.73 | 0.22 |
| Item 4 (soil pollution) | 1.16 | 0.58 | -0.03 | -0.27 | 0.46 | 0.33 | 0.75 | 0.24 |
| Item 5 (animal protection) | 1.48 | 0.70 | -0.95 | -0.39 | 0.56 | 0.42 | 0.74 | 0.23 |
| Item 6 (plant protection) | 1.54 | 0.75 | -1.22 | -0.12 | 0.69 | 0.57 | 0.72 | 0.21 |
| Item 7 (waste disposal) | 1.82 | 0.47 | -2.60 | 6.02 | 0.49 | 0.39 | 0.74 | 0.23 |
| Item 8 (waste classification) | 1.14 | 0.96 | -0.29 | -1.85 | 0.63 | 0.45 | 0.74 | 0.23 |
| Item 9 (water conservation) | 1.77 | 0.53 | -2.20 | 3.85 | 0.57 | 0.47 | 0.73 | 0.22 |
| Item 10 (electricity conservation) | 1.59 | 0.64 | -1.25 | 0.39 | 0.62 | 0.50 | 0.73 | 0.22 |
| Item 11 (paper saving) | 0.88 | 0.75 | 0.20 | -1.21 | 0.39 | 0.22 | 0.76 | 0.25 |

Note: r.raw = correlation between the item and the total score; r.drop = item-total correlation without that item itself when calculating the total score; alpha.drop = the raw Cronbach's alpha if the item has been dropped; average.r.drop = the average inter-item correlation if an item has been dropped.


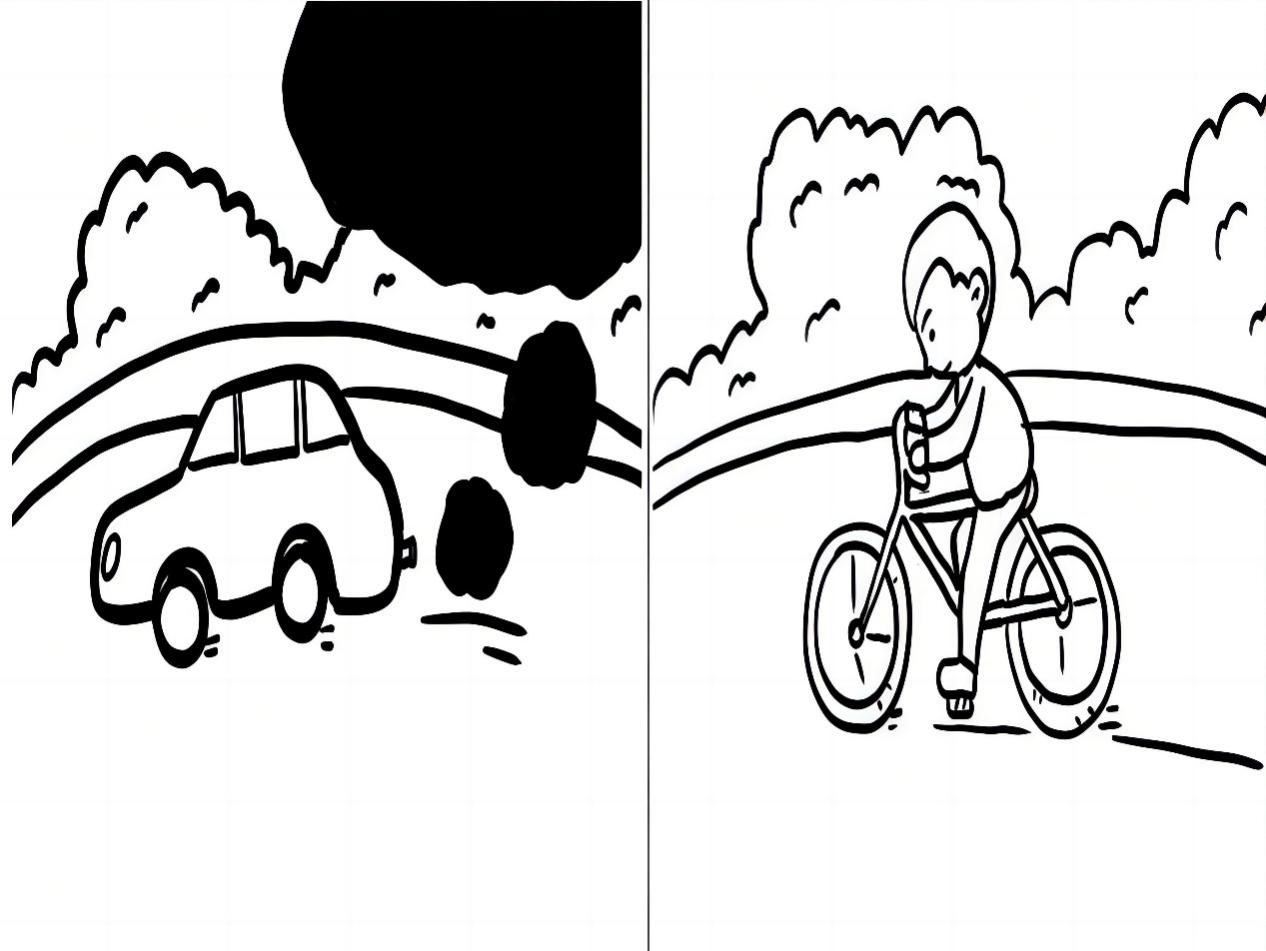


Figure S1. Images for testing pro-environmental attitudes (air pollution). Left panel: Some people like to drive cars when they go out. Right panel: Some people like to ride bikes when they go out.


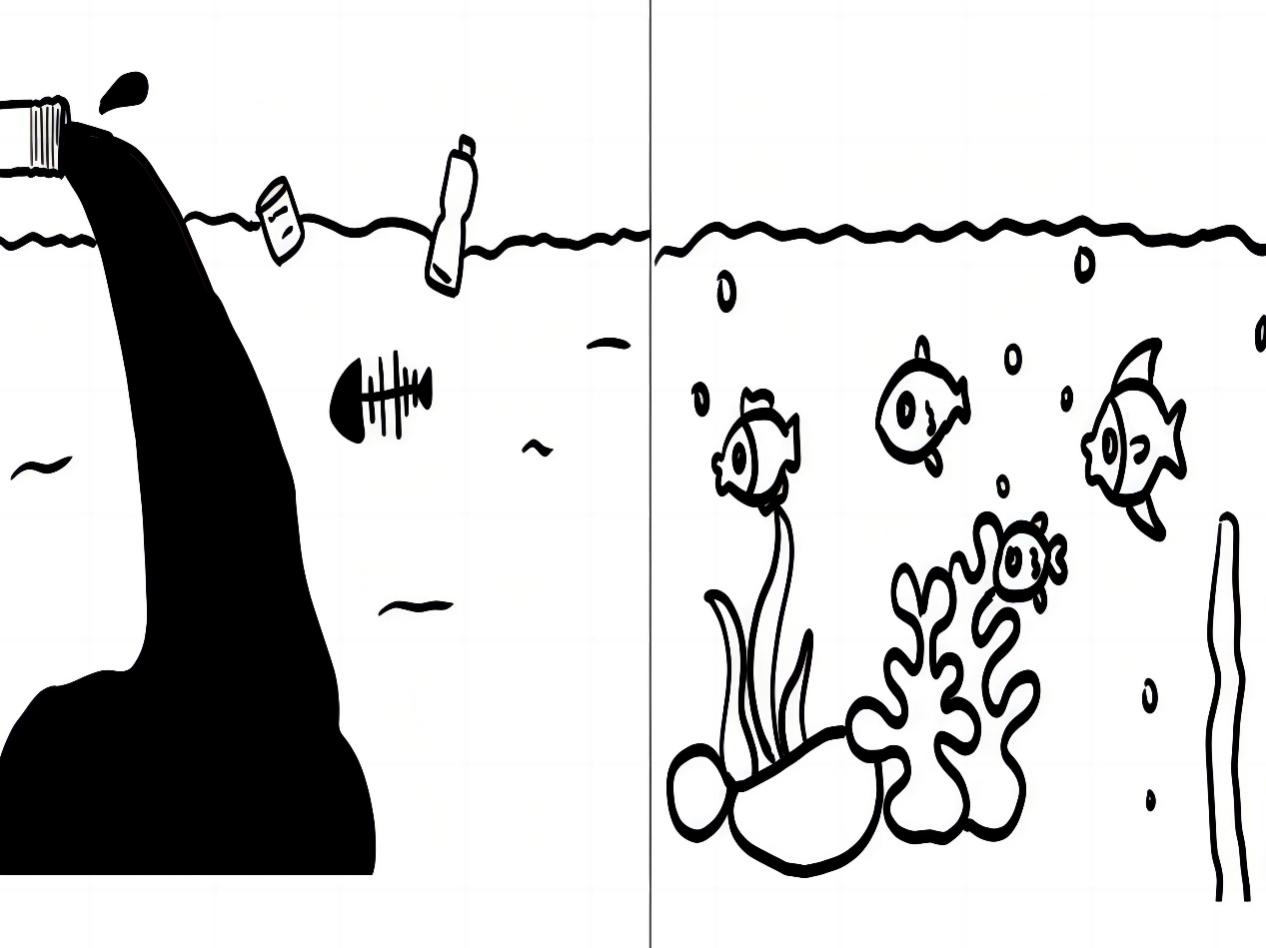


Figure S2. Images for testing pro-environmental attitudes (water pollution). Left panel: Someone discharged sewage into the river, and the water was very dirty. Right panel: No one discharges sewage into the river, and the water is very clean.


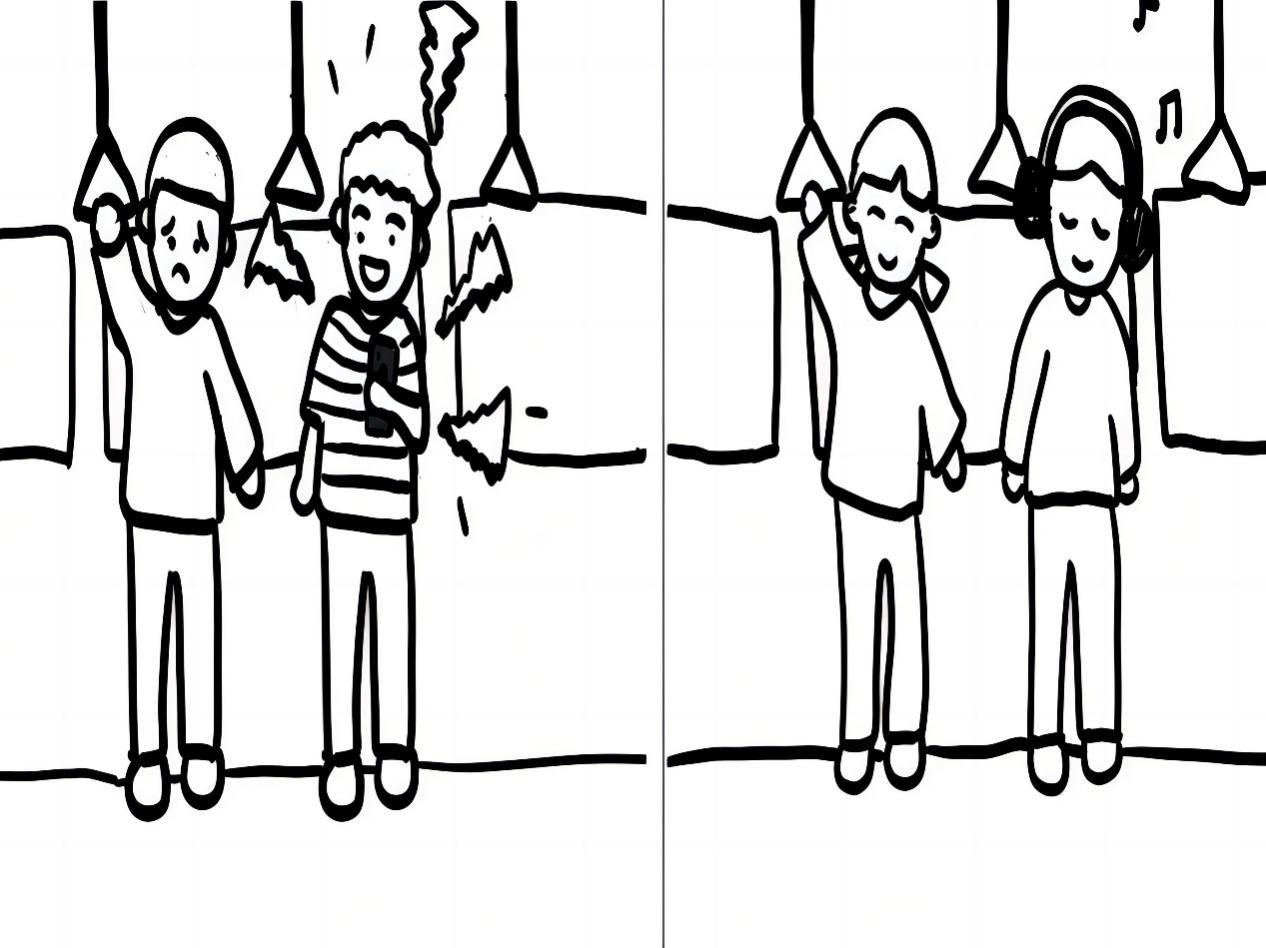


Figure S3. Images for testing pro-environmental attitudes (noise pollution). Left panel: Someone was riding public transportation with their cell phone turned up, disturbing the person next to them. Right panel: Some people ride public transportation with their cell phone volume turned down without disturbing the person next to them.


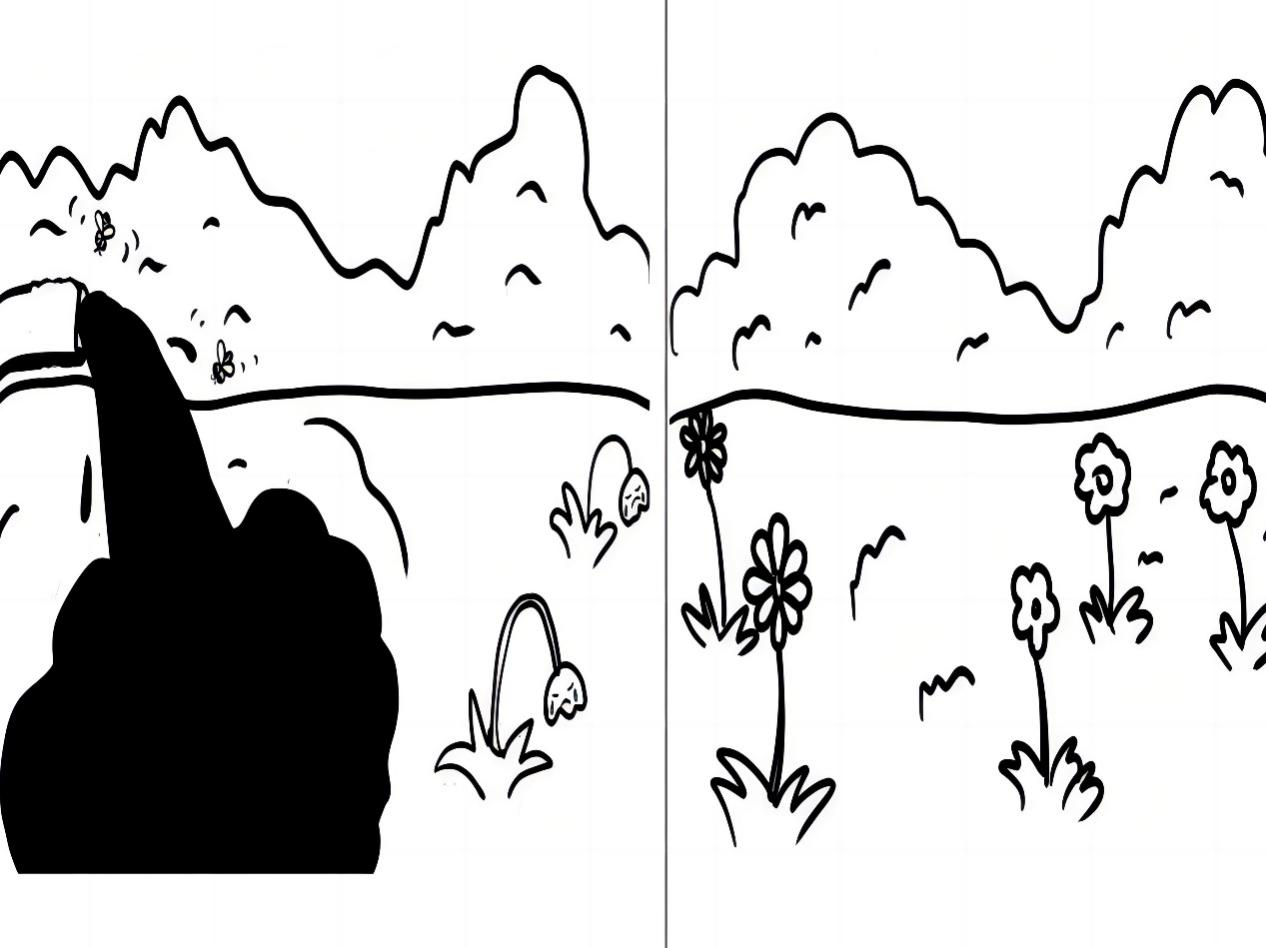


Figure S4. Images for testing pro-environmental attitudes (soil pollution). Left panel: Someone discharged sewage into the soil, and all the flowers wilted. Right panel: No one puts sewage into the soil, and the flowers grow vigorously.


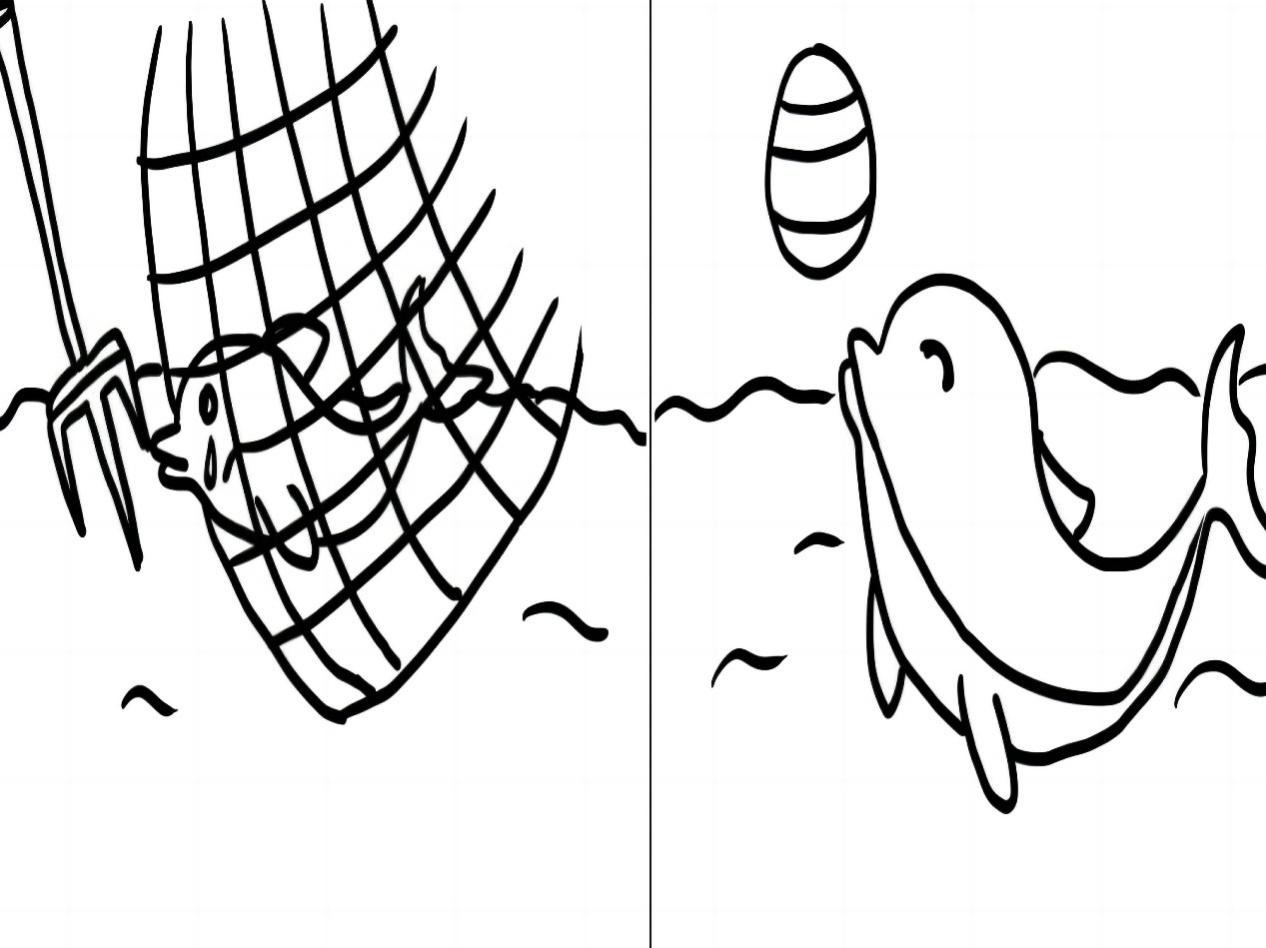


Figure S5. Images for testing pro-environmental attitudes (animal protection). Left panel: People are hunting and killing animals. Right panel: No one hunts or hurts animals.


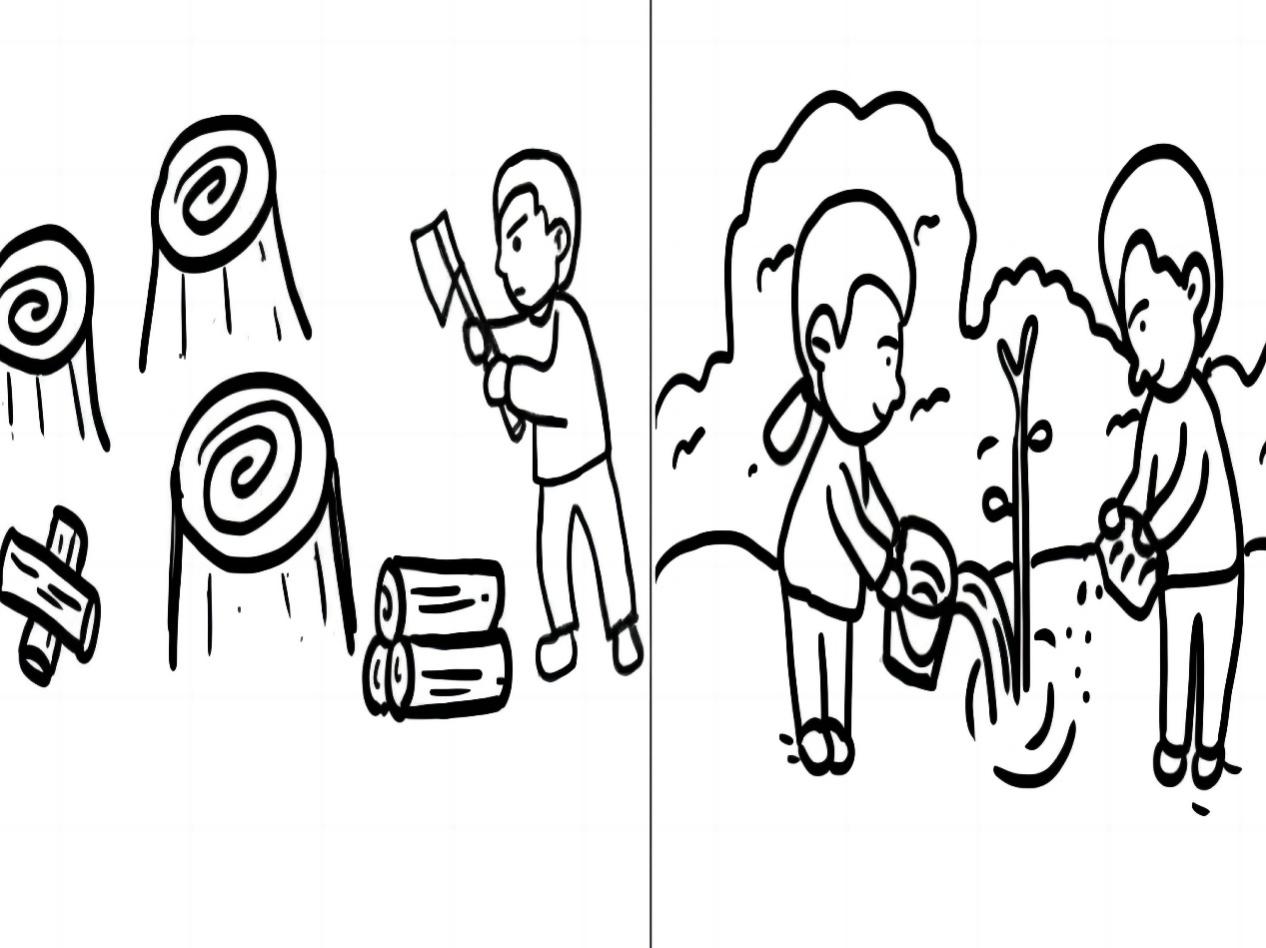


Figure S6. Images for testing pro-environmental attitudes (plant protection). Left panel: Someone is cutting down the trees. Right panel: Someone is watering the trees.


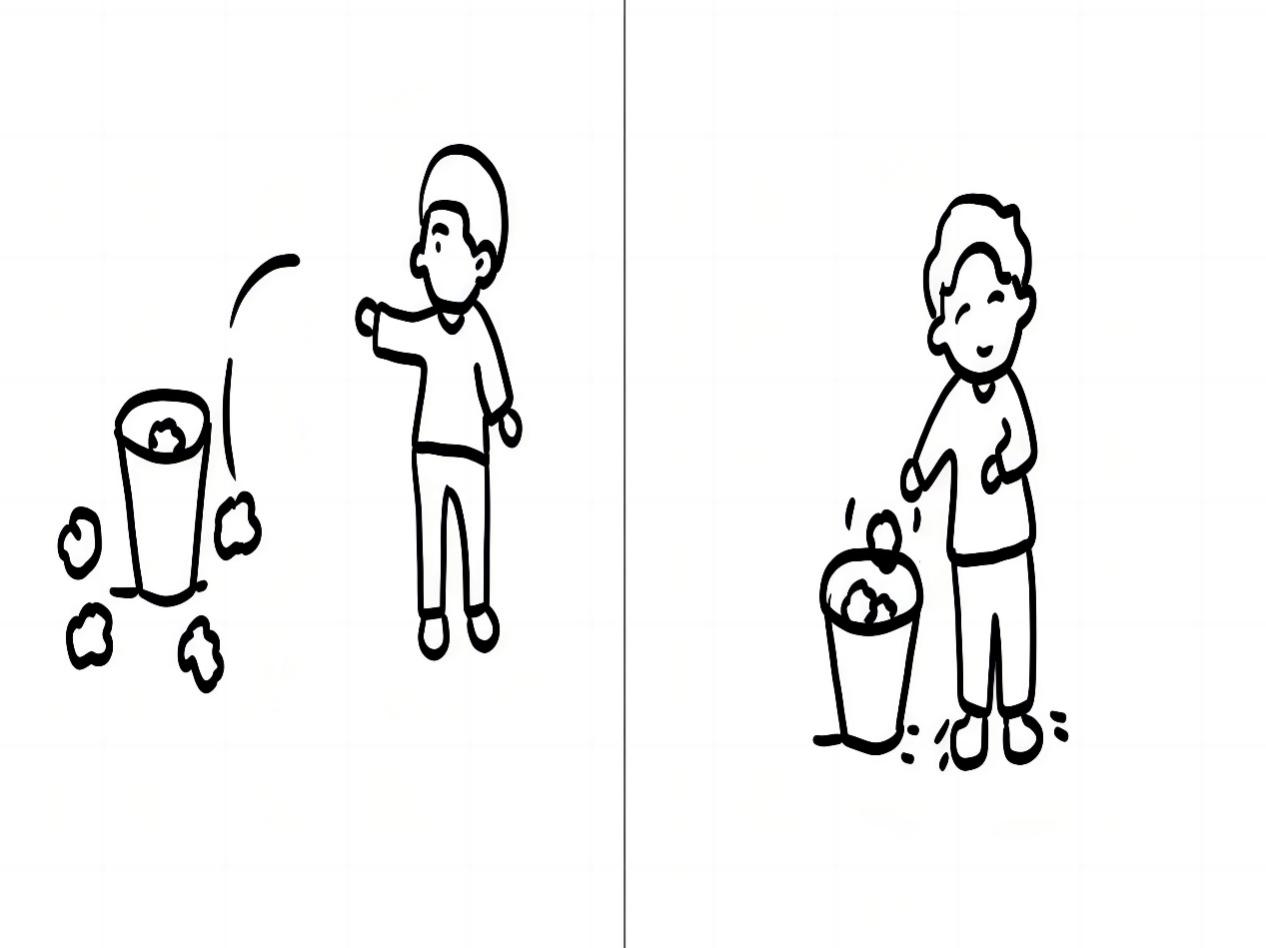


Figure S7. Images for testing pro-environmental attitudes (waste disposal). Left panel: Someone is throwing trash on the ground. Right panel: Someone throws trash in the trash can.


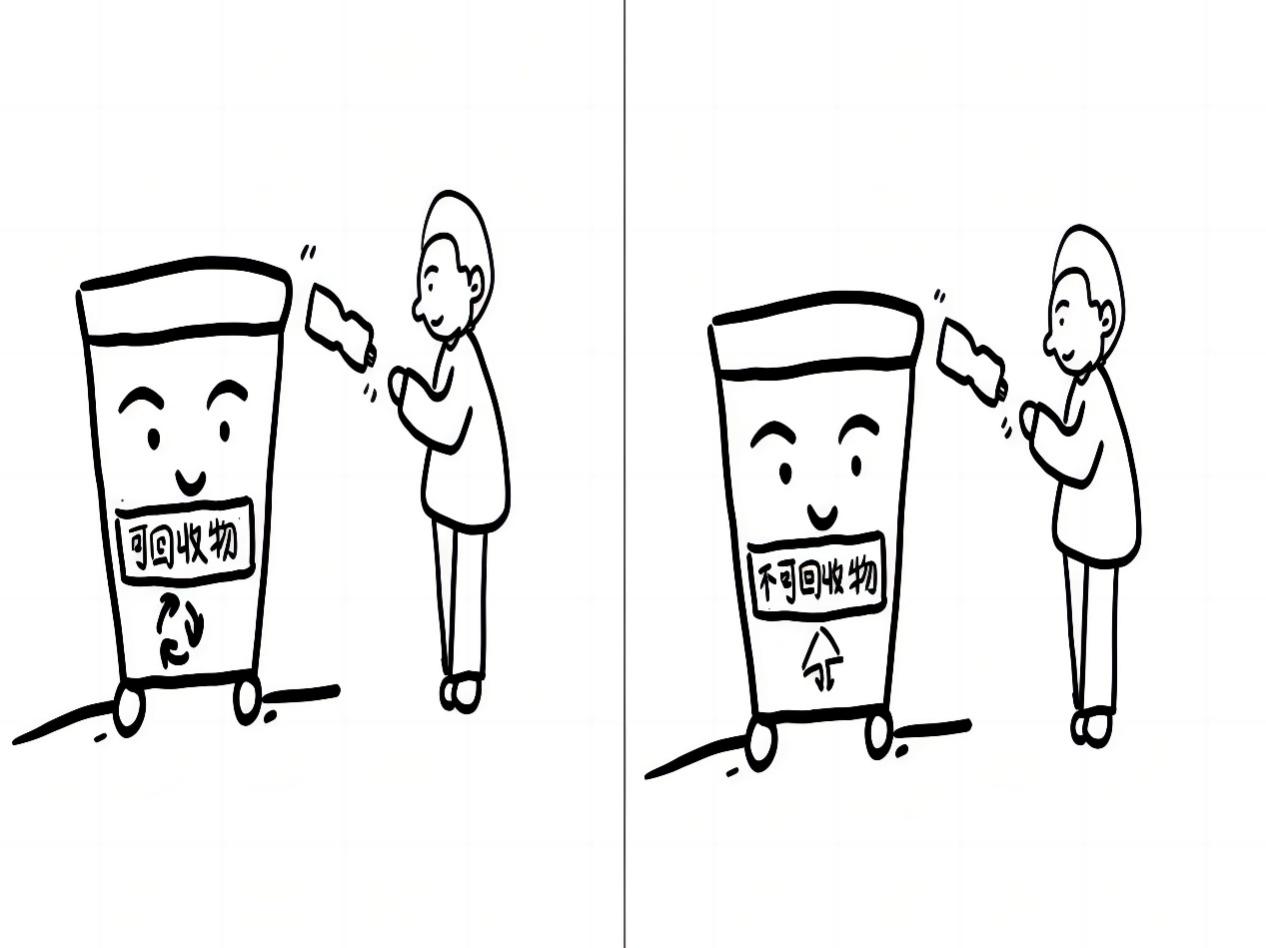
Figure S8. Images for testing pro-environmental attitudes (waste classification). Left panel: Some people throw plastic bottles in non-recyclable trash cans. Right panel: Some people throw plastic bottles in recyclable trash cans.


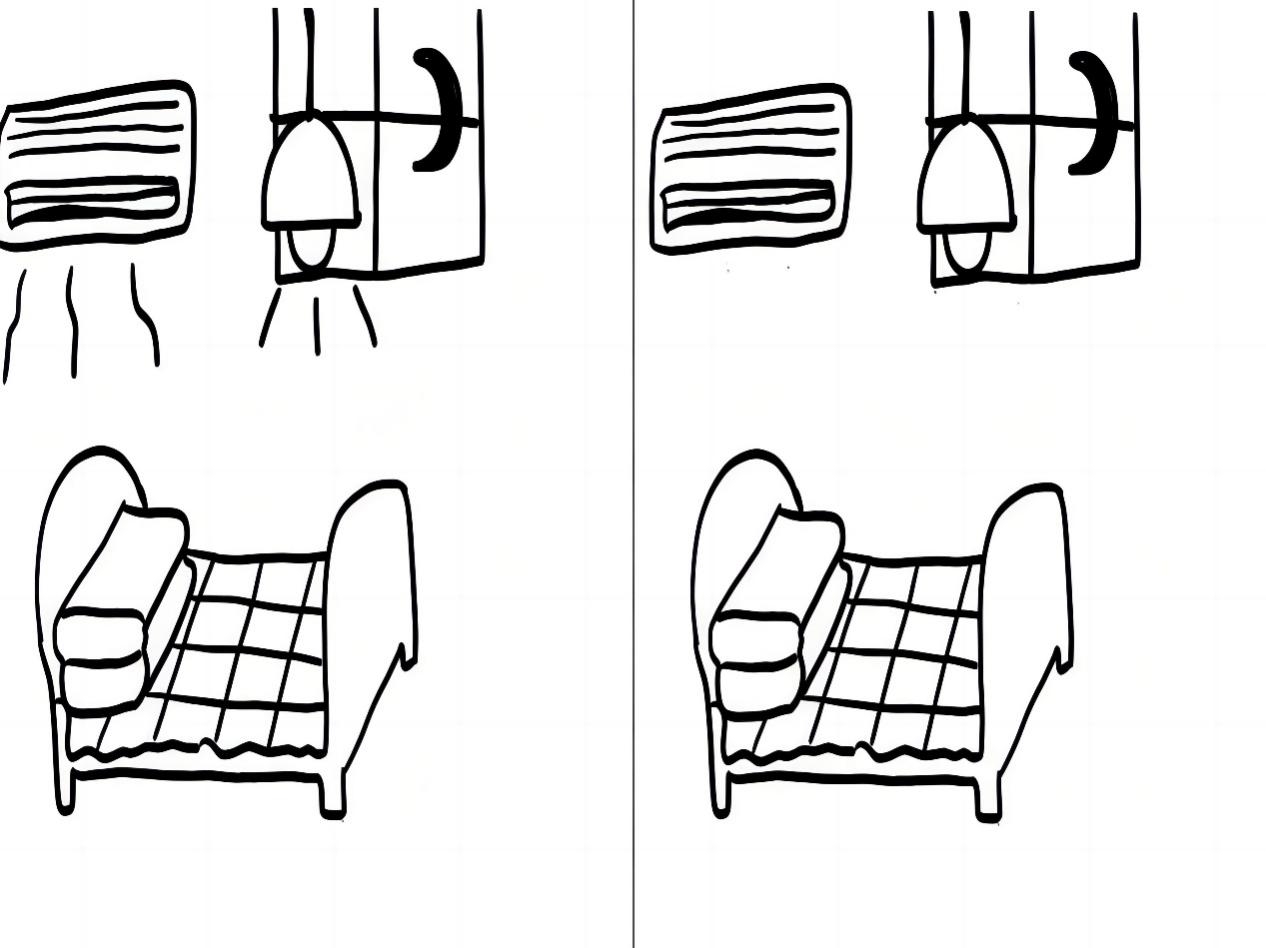
Figure S9. Images for testing pro-environmental attitudes (electricity conservation). Left panel: Someone left their appliances on when they left the house. Right panel: Someone turns off their appliances when they leave the house.


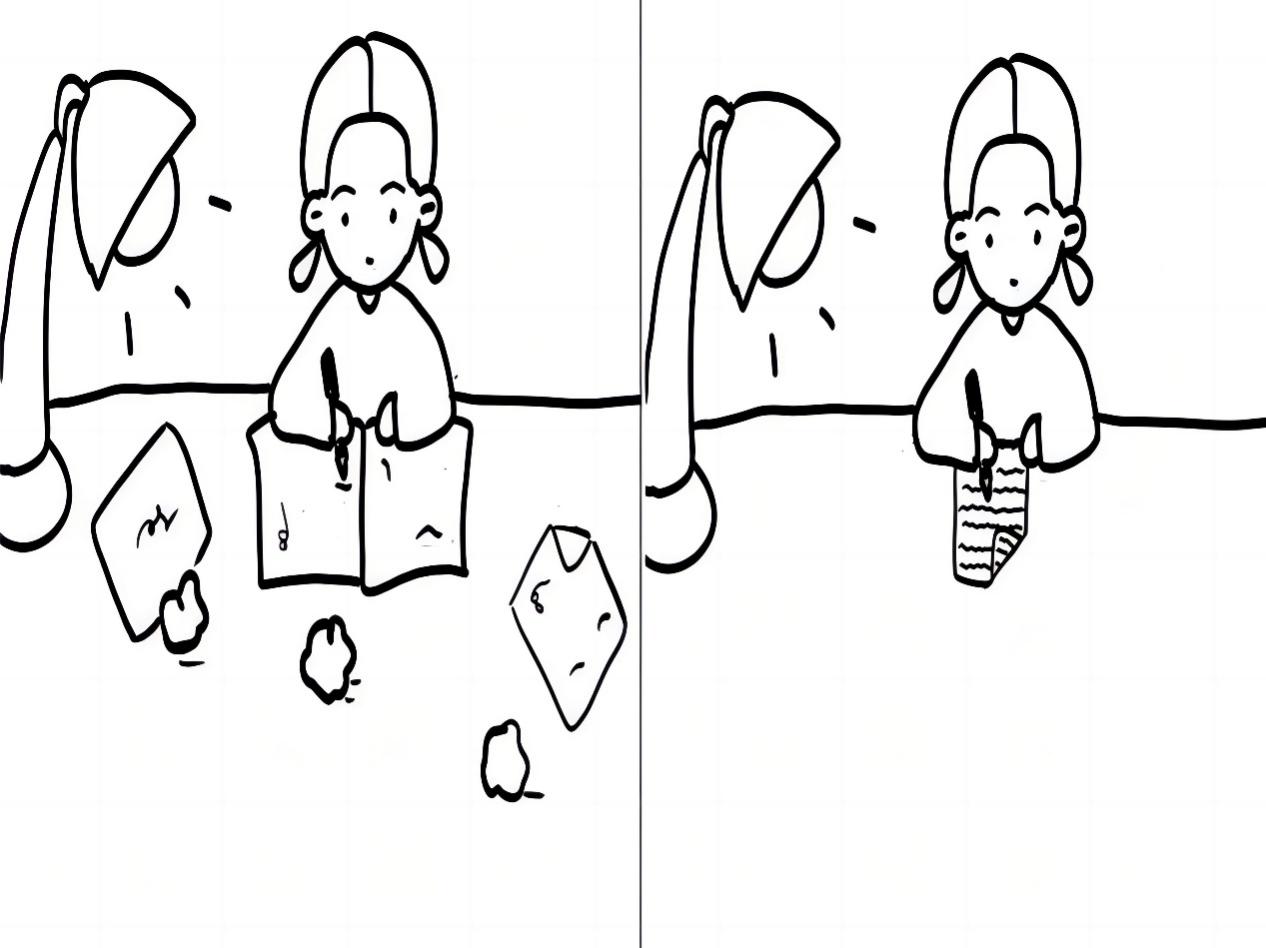
Figure S10. Images for testing pro-environmental attitudes (paper saving). Left panel: Some people write on one side of the paper. Right panel: Some people use both sides of the paper when writing.
